# Supplementary material for: A viral genome packaging ring-ATPase is a flexibly coordinated pentamer
Source: Nat Commun. 2021 Nov 12;12:6548. doi: 10.1038/s41467-021-26800-z (PMC8589836; doi:10.1038/s41467-021-26800-z)
Supplement: Supplementary file 1 — Supplementary Information [file 41467_2021_26800_MOESM1_ESM.pdf]

Supplementary Figures for:

A viral genome packaging ring-ATPase is a flexibly coordinated pentamer

Li Dai[1*], Digvijay Singh[2,3,†*], Suoang Lu[4], Vishal I. Kottadiel[1], Reza Vafabakhsh[4,‡], Marthandan Mahalingam[1], Yann R. Chemla[3-5,**], Taekjip Ha[2-5,**], Venigalla B. Rao[1,**]

[1]Bacteriophage Medical Research Center, Department of Biology, The Catholic University of America, 620 Michigan Ave. N.E., Washington, DC 20064, USA.

[2]Howard Hughes Medical Institute, Department of Biophysics and Biophysical Chemistry**,** Johns Hopkins University School of Medicine, Baltimore, MD 21205, USA.

[3]Center for Biophysics and Quantitative Biology, University of Illinois at Urbana-Champaign, Urbana, Illinois 61801, USA}

[4]Department of Physics, University of Illinois at Urbana-Champaign, Urbana, Illinois 61801, USA.

[5]Center for the Physics of Living Cells, University of Illinois at Urbana-Champaign, Urbana, Illinois 61801, USA.

*Co-first

†Present Address:
Division of Biological Sciences, University of California, San Diego, La Jolla, CA 93093, USA.

‡Present Address:
Department of Molecular Biosciences, Northwestern University, Evanston, IL 60208, USA.

**Correspondence: ychemla@illinois.edu; tjha@jhu.edu; rao@cua.edu

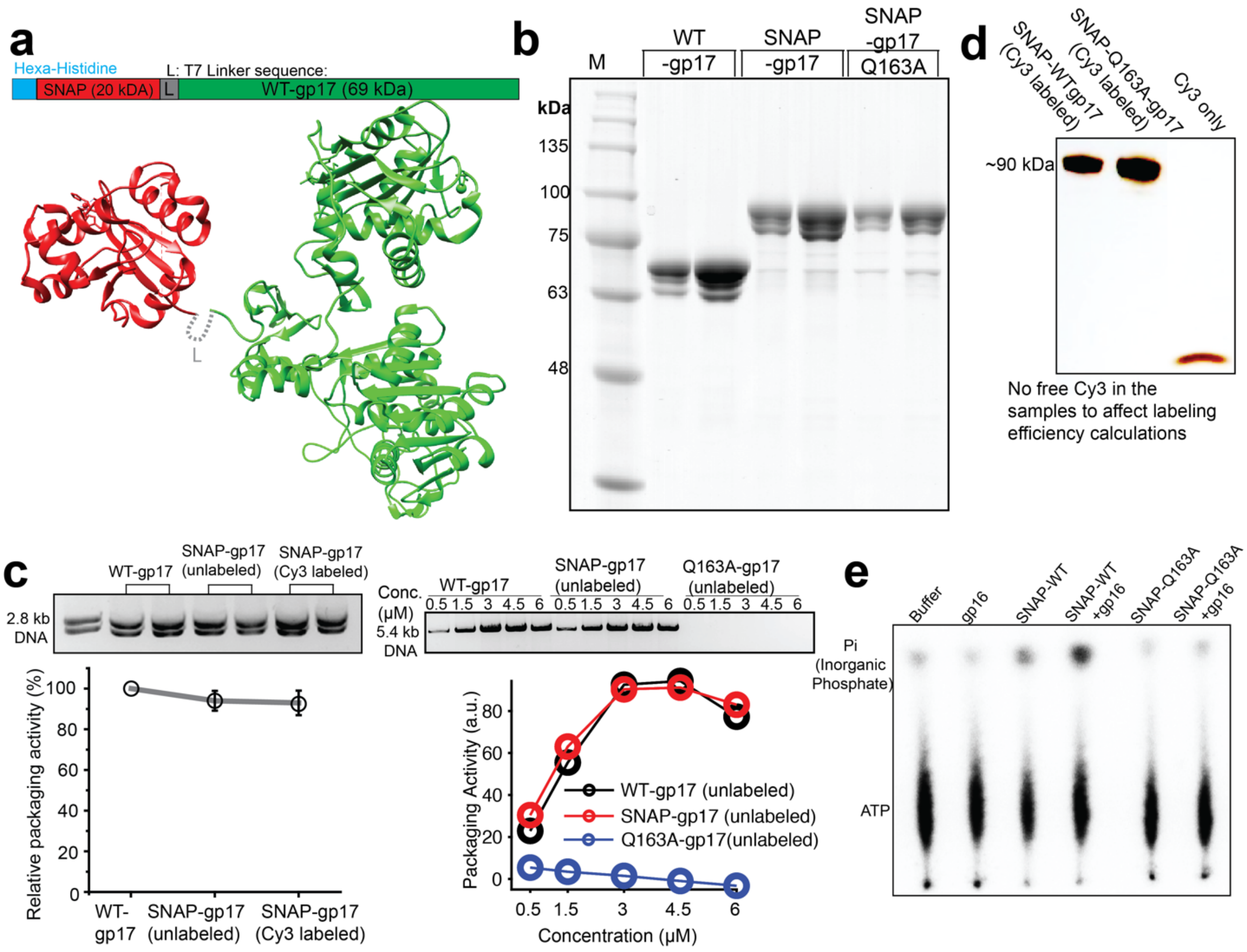

Supplementary Figure 1. SNAP-tag and Cy3 labeling did not impair the motor's activity and biochemical assays showing the activities of the WT and inactive subunit-doped (ISD) motors. (a) Schematic and structural model of gp17 (green) fused to the SNAP-tag (red) at the N-terminal ATPase region through a flexible linker (L) sequence. The model is drawn by the adjacent placement of the structure of SNAP tag (PDB ID: 3kzy) with the structure of gp17 (PDB ID: 3cpe). **(b)** SDS-PAGE of purified protein preparations showing WT gp17, the same fused with SNAP-tag (SNAP-gp17), and inactive subunit fused with SNAP-tag (SNAP-gp17 Q163A). Two different amounts (2X in the right lane vs the left lane) were loaded in each case (the right lane **(c)** Agarose gel image showing the DNA packaging activity of WT-gp17, unlabeled SNAP-gp17, and Cy3-labeled SNAp-gp17 at different concentrations. Shown in the plots are the quantification of the gel bands. These quantifications show that the packaging efficiencies of WT-gp17, unlabeled SNAP-gp17, and Cy3-labeled gp17 are nearly the same confirming that SNAP and Cy3 labeling did not significantly affect packaging activity of the motor **(d).** Excess Cy3 dye was completely removed after the labeling reaction as shown by the lack of any free Cy3 label in the first two lanes**. (e)** The gp16-stimulated ATPase activity of the SNAP-WT gp17 and SNAP-Q163A gp17 mutant was determined using purified proteins (0.5 μM) at a molar ratio of one gp17 monomer to one gp16 oligomer. Note that the weak basal gp17 ATPase activity of WT gp17 (large terminase) is stimulated by gp16 (small terminase), and is impaired in the Q163A gp17 mutant.

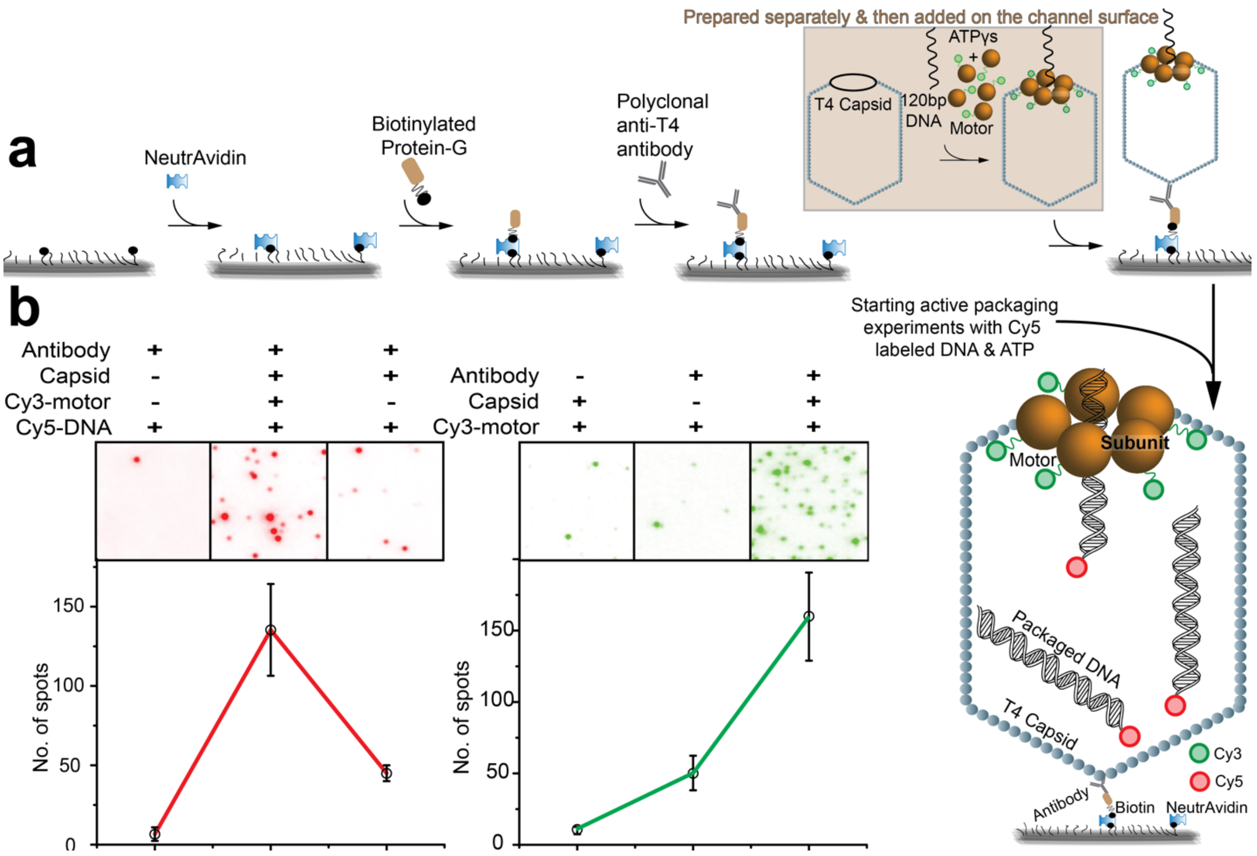

Supplementary Figure 2. Controls showing that all entities of an immobilized packaging motor are required to obtain Cy3 and Cy5 signals in single-molecule assays, validating these assays' authenticity. **(a)** Schematic depicting the steps involved in the reconstitution of the assembled T4 motor for single-molecule fluorescence studies. Homomeric gp17 subunits constituting the motor are labeled with Cy3. (**b**) Individual spots in the Cy3 channel (right) are indicative of surface-immobilized Cy3 labeled motor. Individual spots in the Cy5 channel (left) are indicative of the DNA packaged into the capsid by the surface-immobilized Cy3 labeled motor (middle panel). In the control panel (left panel) lacking capsid and motor, rare non-specific background spots are seen. In another control lacking the motor, only a few spots are seen due to the association of DNA to the capsid portal in the absence of the motor (right panel).

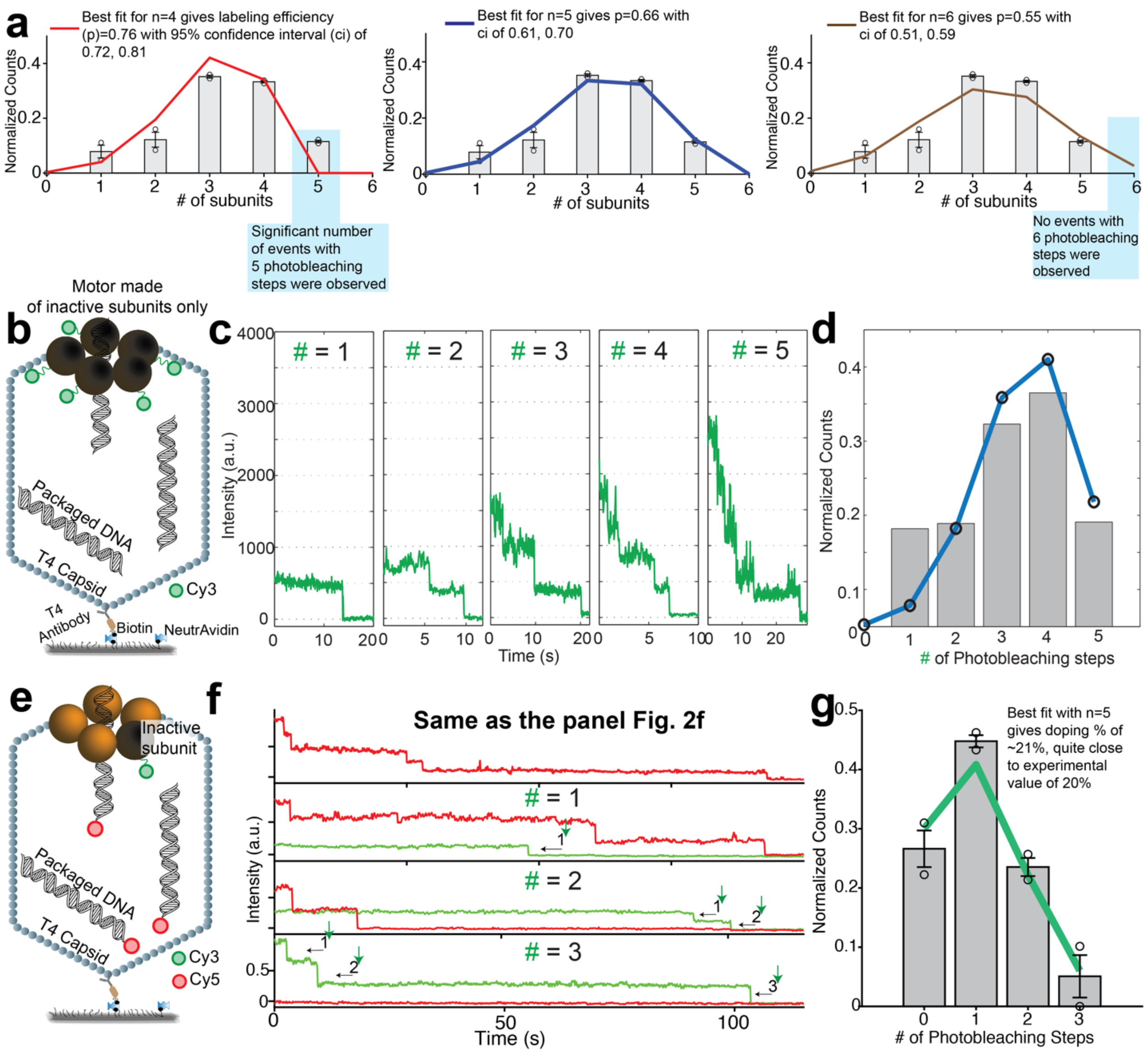

Supplementary Figure 3. Assessing the stoichiometry of the WT motor and propensity of inactive subunits in proper motor assembly. (a) Quality of fits to the distribution of Cy3 photobleaching steps assuming different putative stoichiometries of the motor. (**b-d**) Inactive subunits also assemble to form pentameric motor. (b) Schematic of assay similar to that in Figure 1a, but using Cy3 labeled inactive subunits only. (c) Representative single-molecule fluorescence trajectories, showing different number of Cy3 photobleaching steps. (d) Distribution of the number Cy3 photobleaching steps pooled from multiple trajectories. With the Cy3 labeling efficiency of the inactive subunit at 68%, the best binomial fit (blue curve) yielded the stoichiometry of 5, confirming that inactive subunits can assemble into a pentameric motor complex, same as WT motor subunits. (**e-g**) Inactive subunits and WT subunits have the same propensity of assembly into the pentameric motor. (e) Schematic of assay similar that in Figure 1a, but using motors doped with 20% of Cy3-labeled inactive subunits, as in the experiments described in Fig. 2f and Fig. 3. (f) Representative single-molecule fluorescence trajectories, showing different number of Cy3 photobleaching steps. (g) Distribution of the number of Cy3 photobleaching steps pooled from multiple trajectories and fit. The binomial fit to the distribution gives the doping % of 21%, close to the experimental value of 20%, showing that inactive subunits have the same propensity as WT subunits to assemble into pentameric motors. For all experiments with error bars, ≥ 2 replicate & reproducible

experiments were performed to get statistics of mean and standard deviation. Values represent mean. Error bars throughout represent std.

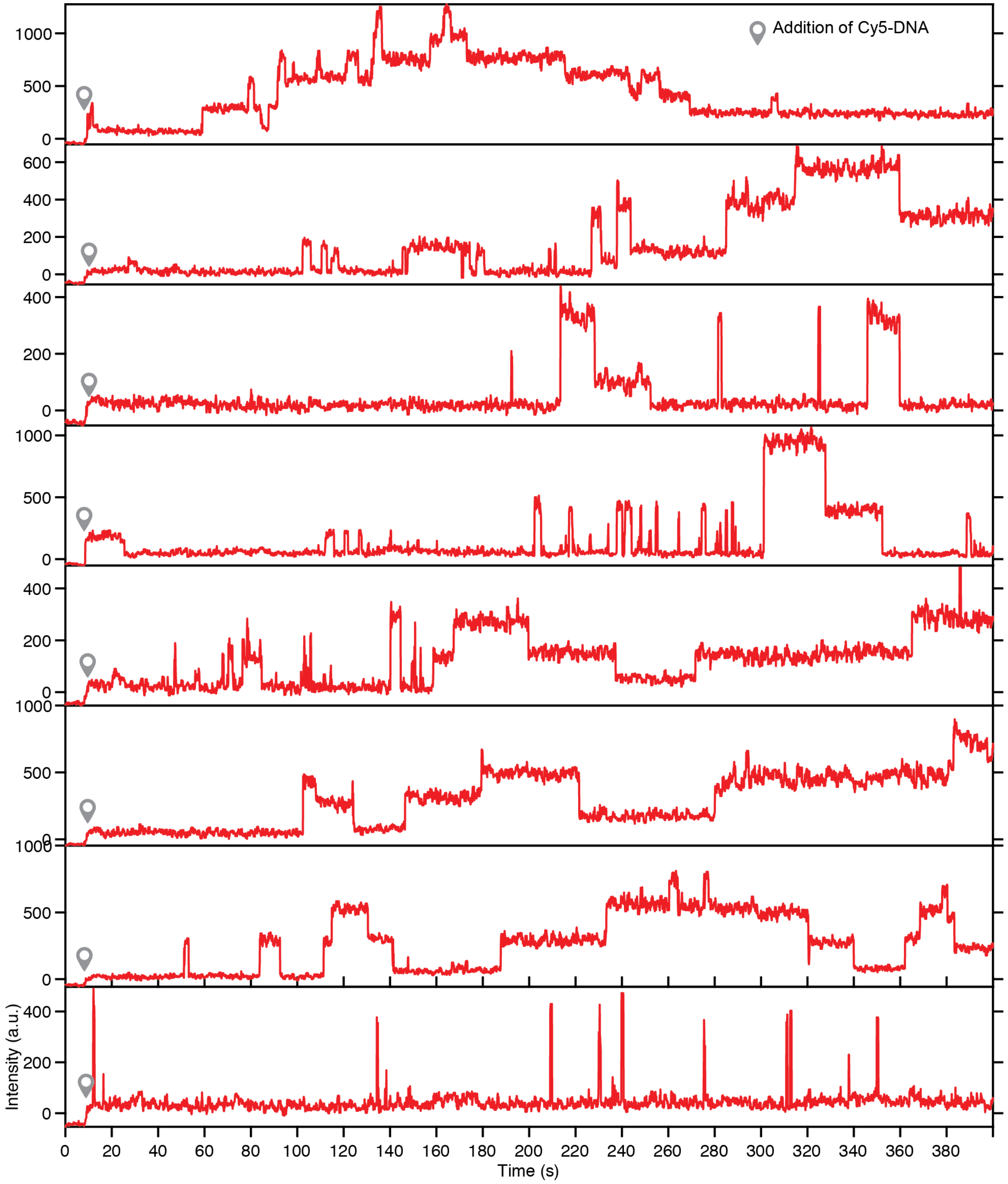

Supplementary Figure 4. Representative trajectories showing the real-time DNA engagements by WT motors with no inactive subunits.

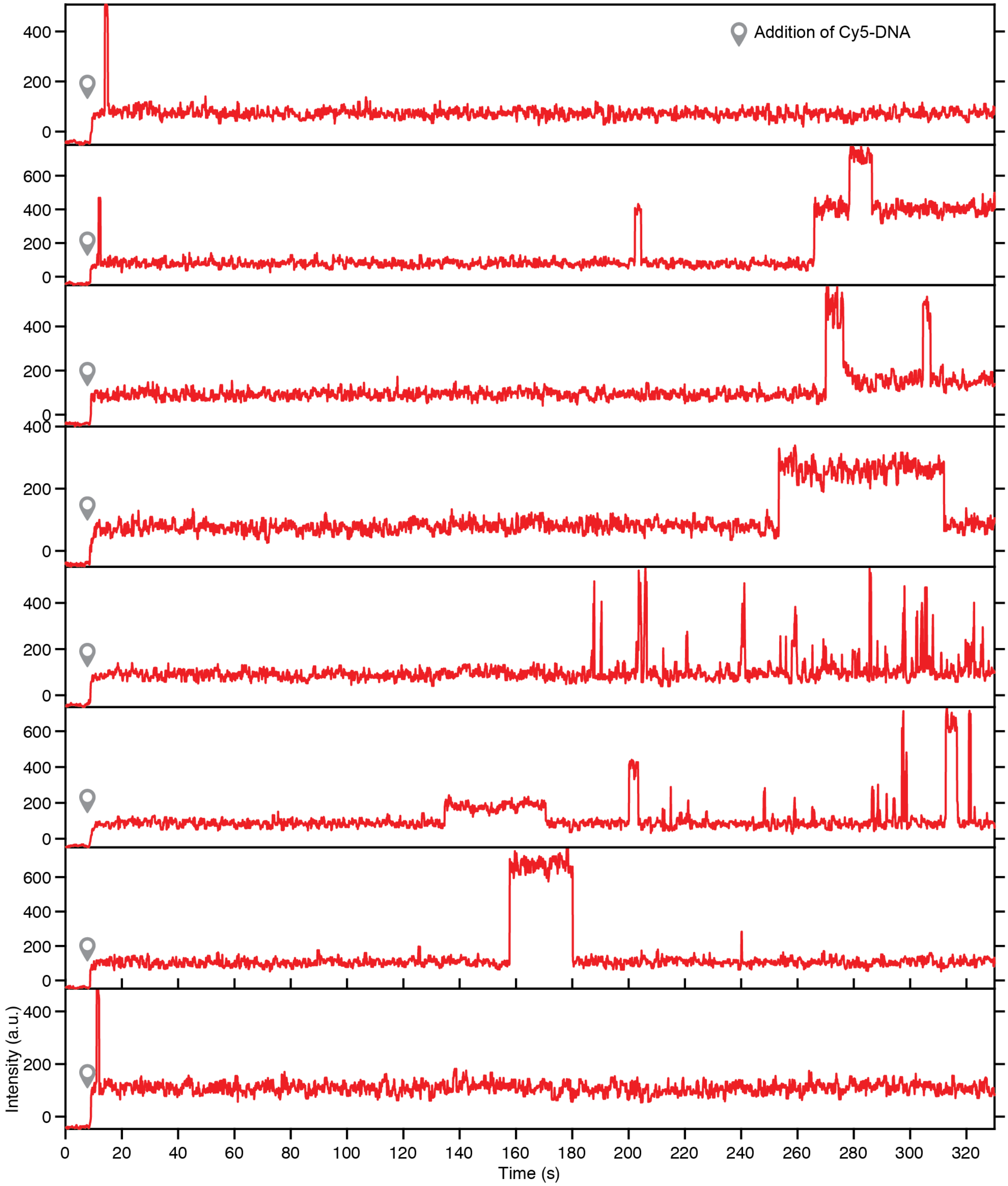

Supplementary Figure 5. Representative trajectories showing the real-time DNA engagements by ISD motors with 1 inactive subunit.

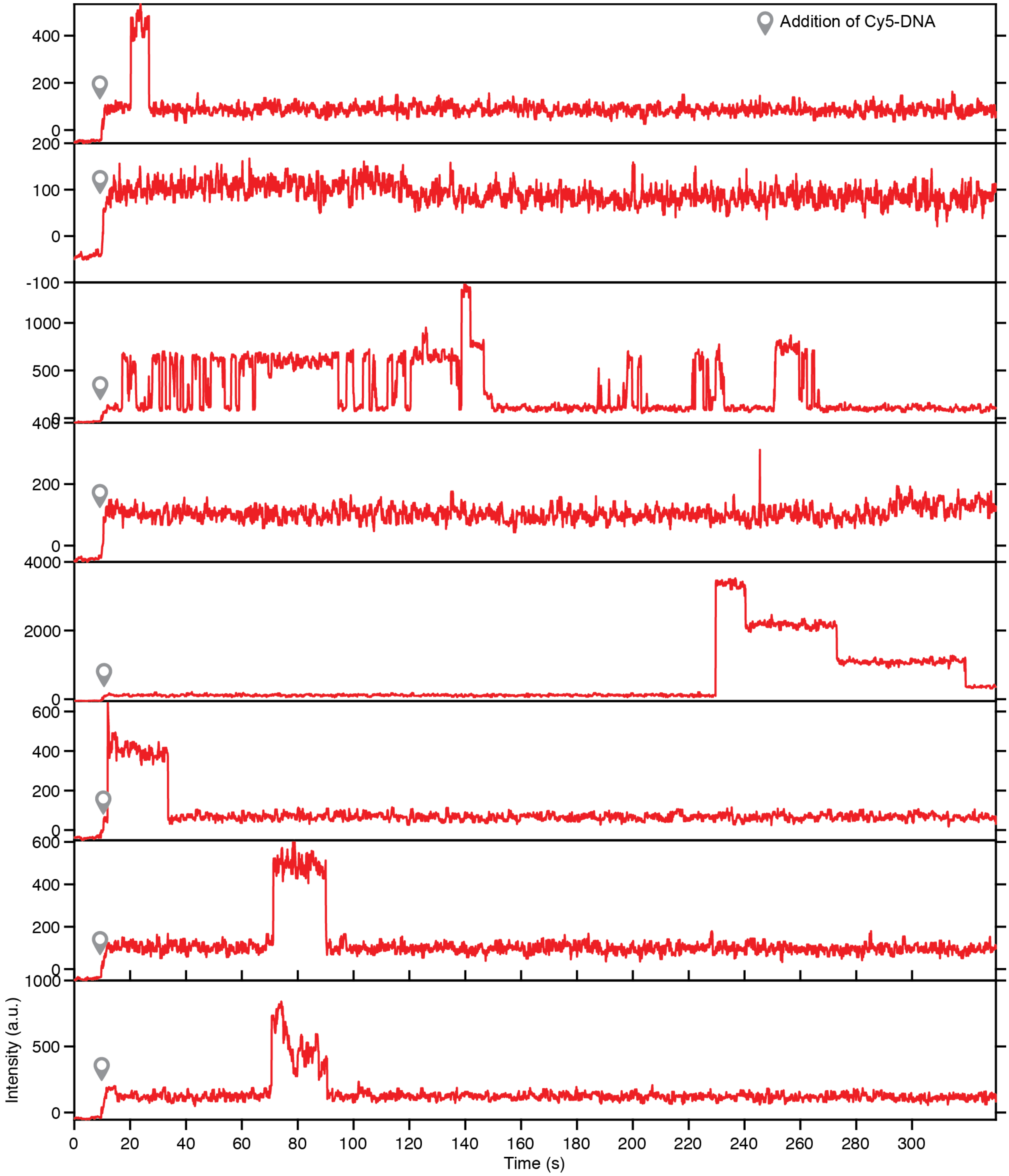

Supplementary Figure 6. Representative trajectories showing the real-time DNA engagements by ISD motors with 2 inactive subunits.

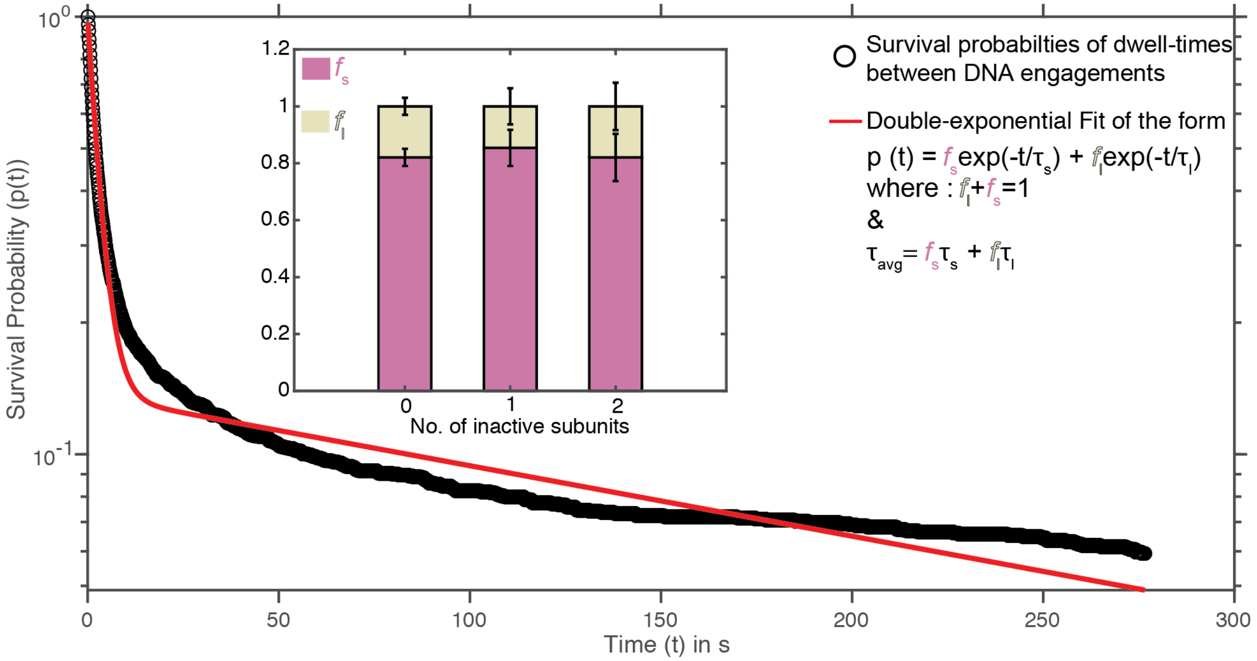

Supplementary Figure 7. Fitting the survival probability distribution of the dwell-times between the DNA engagements from a replicate experiment with the ISD motor containing 1 inactive subunit. Inset shows f_s and f_l values for different motors. For all experiments with error bars, 2-5 replicate & reproducible experiments were performed to get statistics of mean and standard deviation. Values represent mean. Error bars throughout represent std.

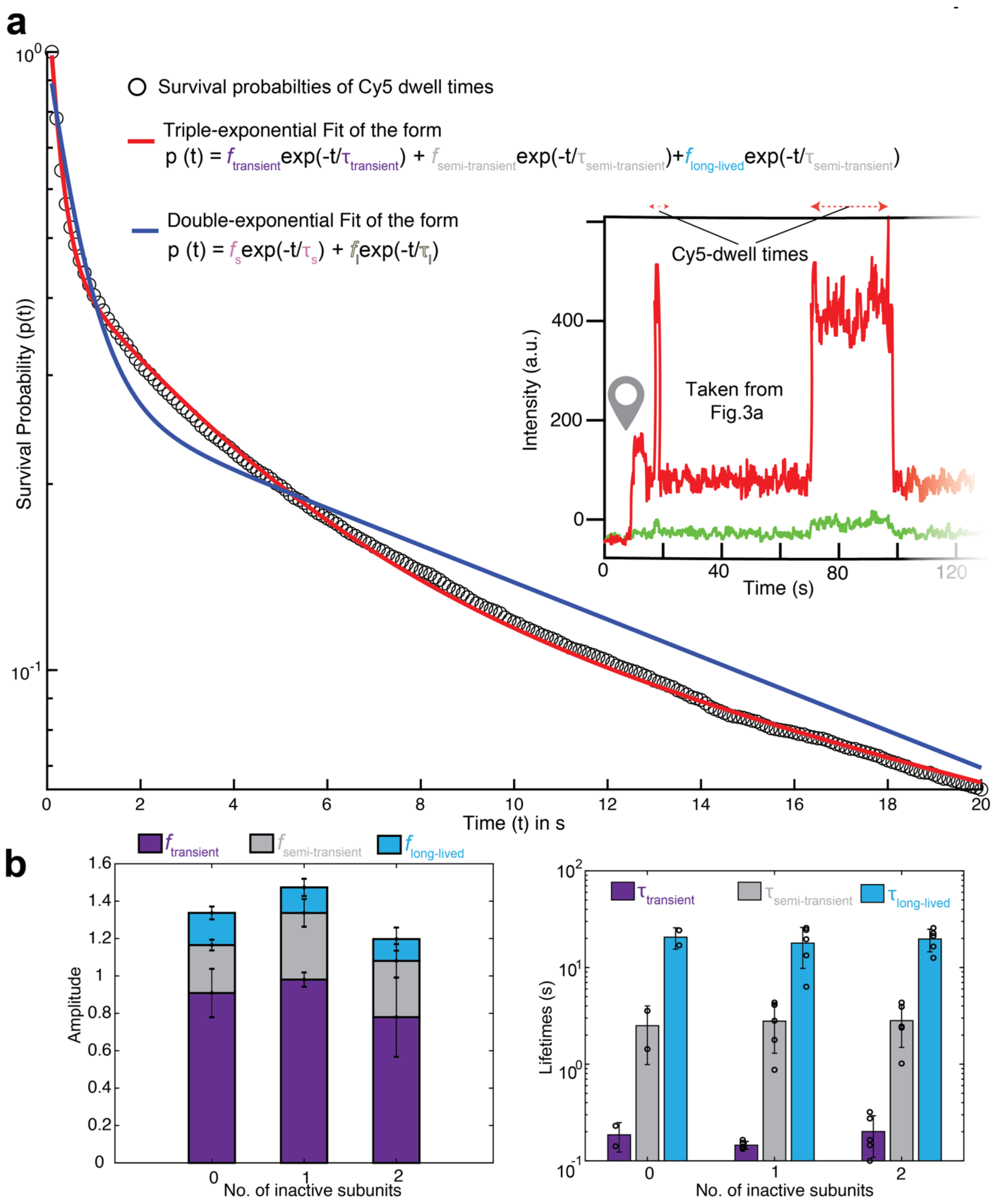

Supplementary Figure 8. Distributions of dwell times of DNA engagements. (a) Survival probability distribution of the dwell times of the DNA engagements from a replicate experiment with the WT motor, and fits to a double- (blue) and triple-exponential functions (red). The inset shows representative dwell times of Cy5 signal, indicating DNA engagements, from a single-molecule fluorescence trajectory. (**b**) Amplitudes and lifetimes from the triple-exponential fits to the distribution of dwell times of DNA engagements for motors with 0, 1, and 2 inactive subunits. For all experiments with error bars, 2-5

replicate & reproducible experiments were performed to get statistics of mean and standard deviation. Values represent mean. Error bars throughout represent std.

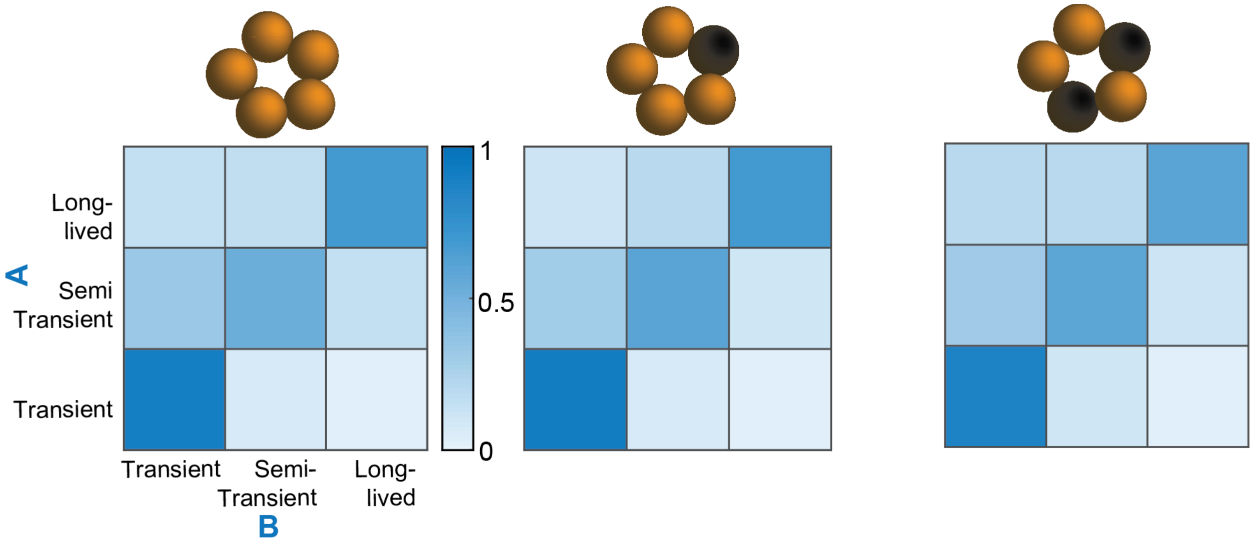

Supplementary Figure 9. DNA engagements of same type tend to cluster together.
Transition probability matrices showing the probability of observing engagement B followed by engagement A of a particular type for motors with 0-2 inactive subunits.

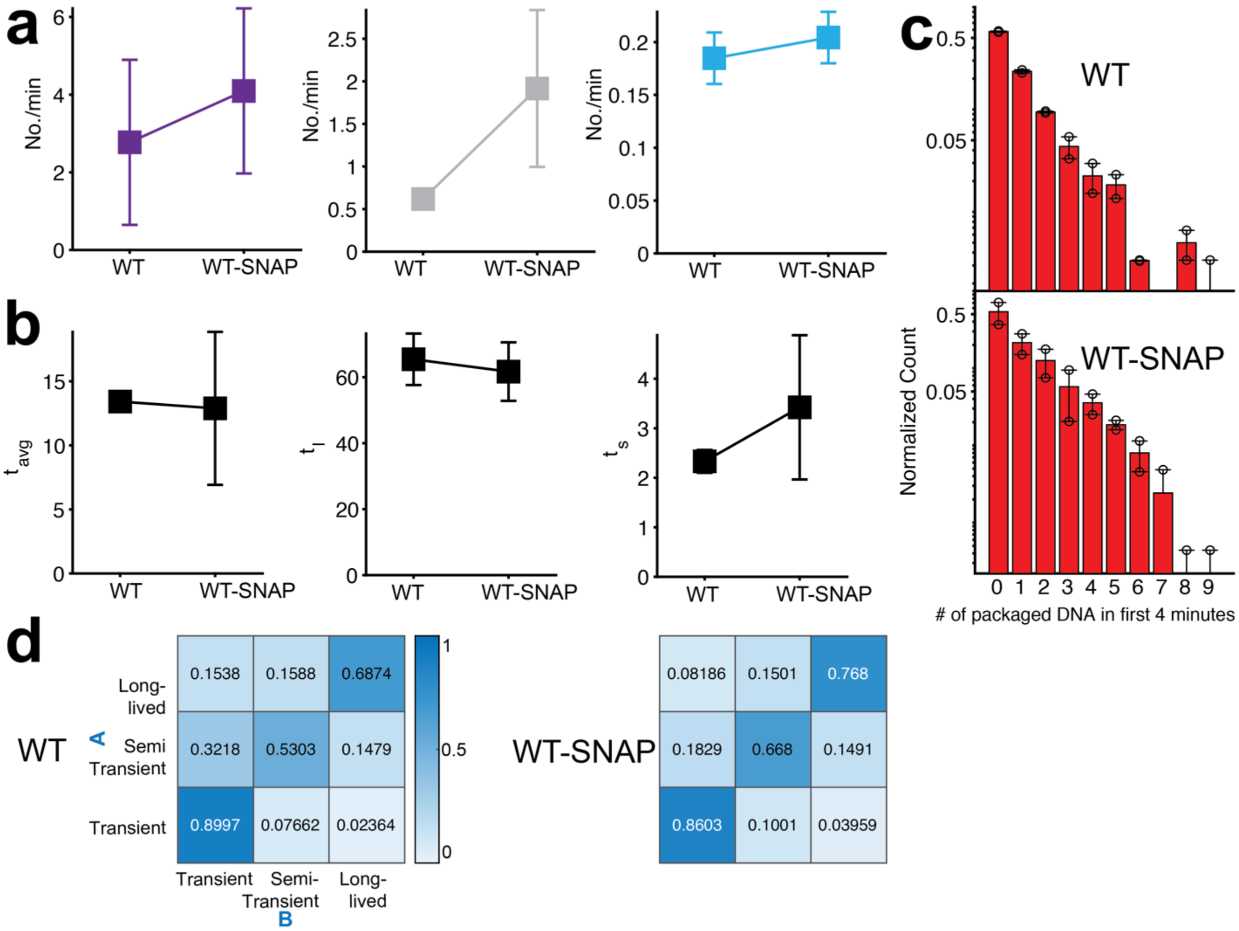

Supplementary Figure 10. Detailed comparisons of DNA engagement behavior between WT motor and WT motor with SNAP-tags show that SNAP-tag(s) do not affect DNA engagements. (a) Number of transient/semi-transient and long-lived DNA engagements per minute for WT motor and WT with SNAP-tags. **(b)** Average time (τ_{avg}) between DNA engagements for for WT motor and WT with SNAP-tags. (left). Lifetimes of DNA engagements occurring in short bursts (τ_s) (middle). Lifetimes of DNA engagements occurring in long-lived delays (τ_l) (right). τ_{avg} is an amplitude-weighted average of τ_s and τ_l. **(c)** Distribution of the #packaged DNA in the first 4 minutes for motors with different inactive subunits. **(d)** Transition probability matrix showing the probability of observing engagement B followed by engagement A of a particular type. For all experiments with error bars, 2-5 replicate & reproducible experiments were performed to get statistics of mean and standard deviation. Values represent mean. Error bars throughout represent std.

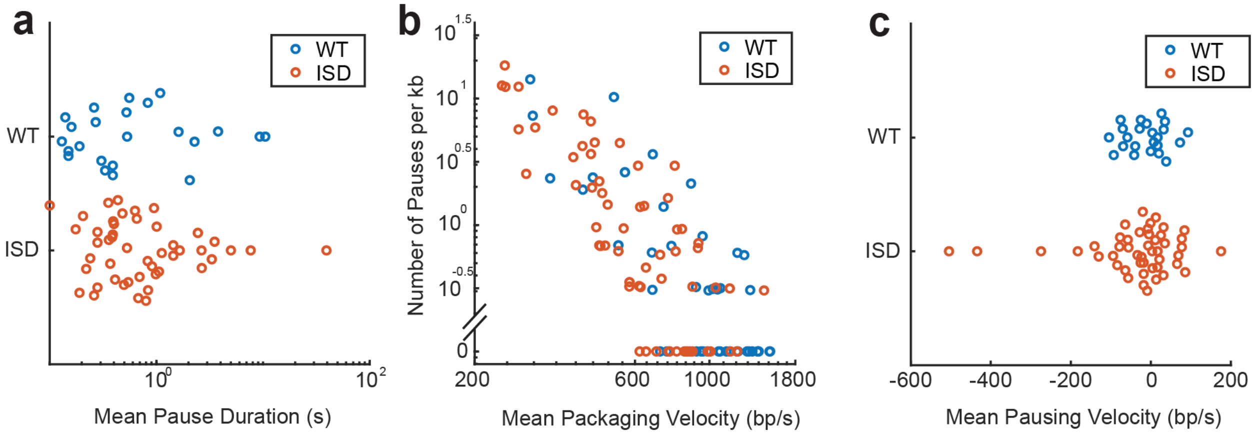

Supplementary Figure 11. Packaging dynamics of WT and ISD motors determined by optical trap assay. **(a)** Distribution of mean pause duration of WT (blue) and ISD motors (orange) which encountered pause(s). **(b)** Distribution of mean packaging velocity vs. pause frequency for each packaging motor, which are negatively correlated. **(c)** Distribution of mean pausing velocity of WT (blue) and ISD motors (orange) which encountered pause(s). The pausing velocity is the velocity during the pause, during which the motor may or may not be unpackaging.

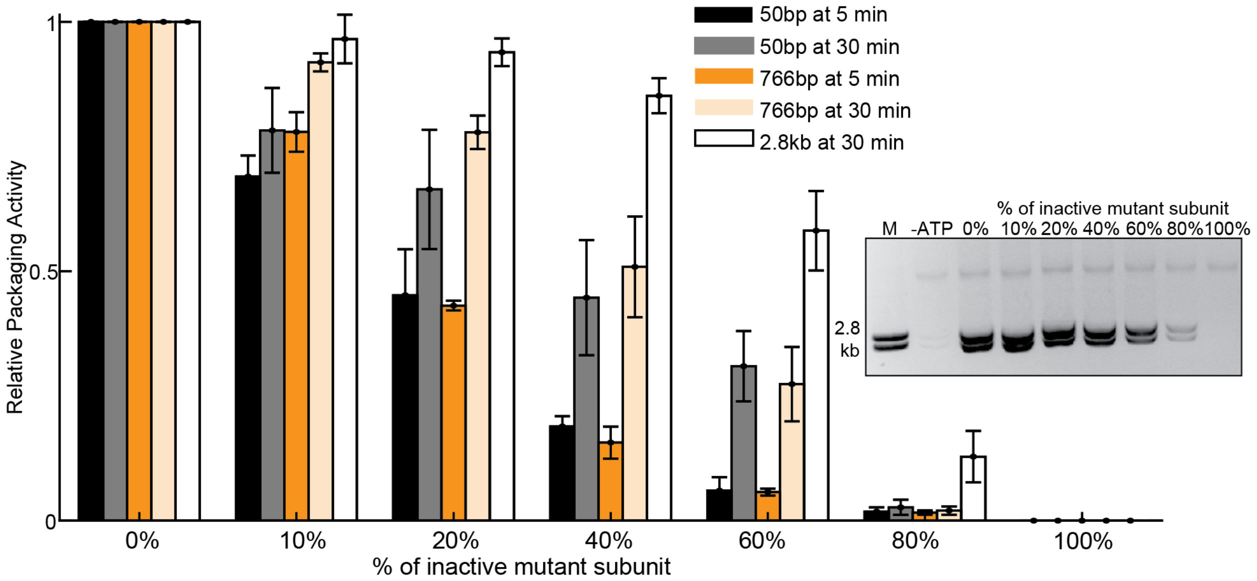

Supplementary Figure 12. DNA packaging activity for DNA of different lengths and thus of different diffusion coefficients shows that DNA with lower diffusion coefficients are less affected by the inactive subunits. Normalized packaging activity for WT and WT mixed with mutant in different ratios. A mixture of 50 bp, 766 bp and 2.8 kb DNAs were used for packaging for 5 min or 30 min incubation times. The inset shows a representative in vitro bulk packaging assay using the 2.8 kb DNA substrate by increasing the percentage of the inactive subunit and giving sufficient packaging time, 30min. Normalized packaging activity for WT and WT mixed with mutant in different ratios. Various length DNAs; 50 bp, 766 bp and 2.8 kb DNAs were used for packaging, for 5 min and 30 min incubation times. For all experiments with error bars, ≥ 2 replicate & reproducible experiments were performed to get statistics of mean and standard deviation. Values represent mean. Error bars throughout represent std.

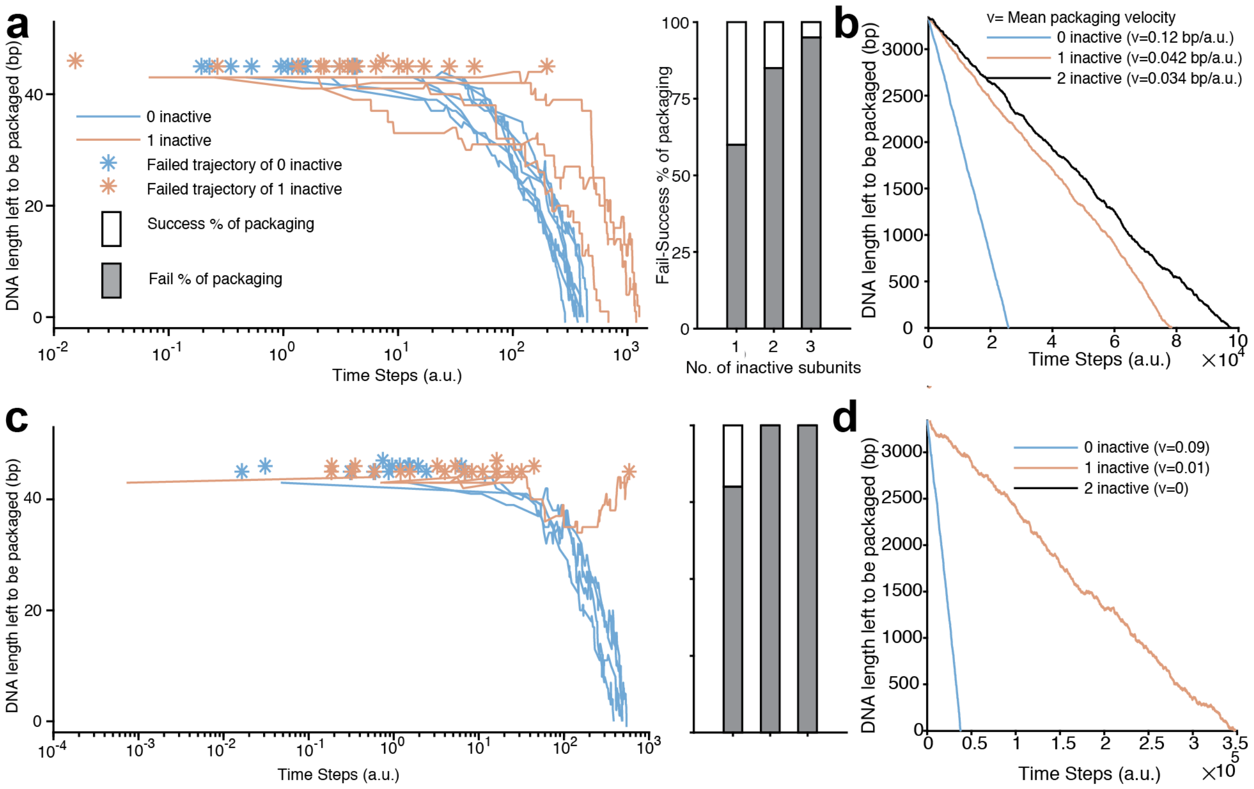

Supplementary Figure 13. Monte Carlo simulations, based on the model described in Figure 5 were conducted to model the: (a) Packaging of 45-bp long DNA, similar to the experiments described in Figure 3. Twenty trajectories representing the DNA's unpackaged length, each for the motor with 0 and 1 inactive subunits, are in the left plot. These simulations highlight how motors, especially with inactive subunit, can begin packaging but ultimately fail in full encapsidation of the DNA. On the right is a bar graph showing the % of successful and failed packaging trajectories. **(b)** Packaging of ~3400 bp long DNA, similar to the experiments described in Figure 4. The y-axis represents the DNA's unpackaged length, similar to the DNA extension reported in the optical trap experiments' representation. The x-axis represents the time-steps in arbitrary units (a.u.). **(c-d)** The same simulation, but with average quanta of slips being twice as high as it was for simulations shown in (a-b). The difference between WT motor, motor with 1 and 2 inactive subunits increase significantly. E.g., the difference in mean packaging velocity across motor with different number of inactive subunits, shown in (d), increases multi-fold. This velocity becomes zero for motor with 2 inactive subunits, as the DNA slips out of the motor completely and thus its trajectory is not shown.

In our experiments, we did not observe a marked difference between motor with 1 and 2 inactive subunits. With the simulation, we learned that the difference between the motor with 1 and 2 inactive subunits is minimal when the quanta of DNA slips at each pause & slip event is small. Therefore, this simulation suggests an important rationalization, i.e., despite motors with 2 inactive subunits encountering the inactive subunits twice more than the motors with 1 inactive subunit, the overall difference in packaging behavior between the two motors is minimal when the average slip length of the DNA during pause & slip is low.

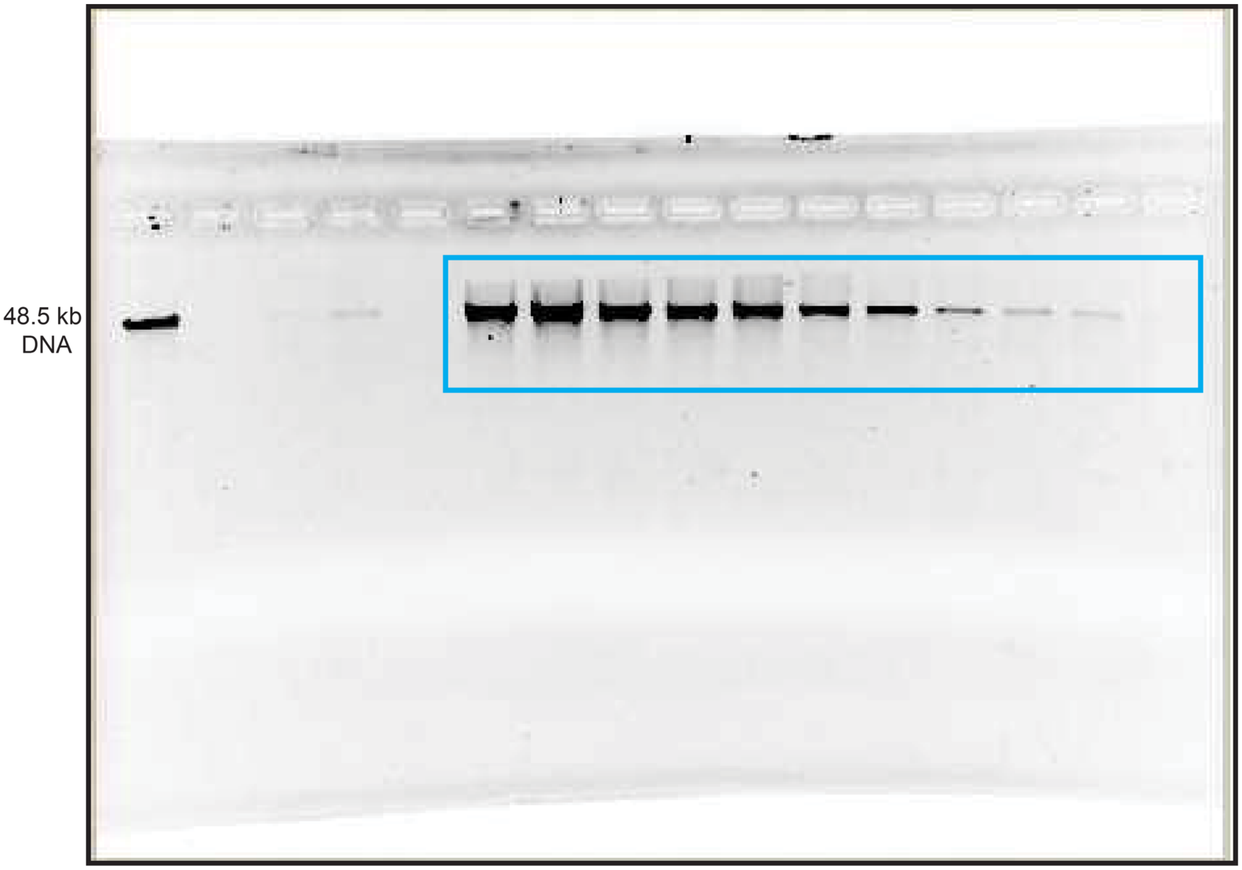

Supplementary Figure 14. Uncut image of the gel shown in Fig. 2b. The region shown in Fig. 2b is highlighted in cyan.

Supplementary Table 1. Sequences of oligos and primers

Entity	Sequence
Cy5-labeled 45-bp DNA oligonucleotide	5′-CA/iCy5/CTCGTCCAGCAGATAGAAGTCACAGCGGATCCTATAGACAGAG-3′
Primers for SNAP -gp17	Forward1: 5'-CCACTGCAGAAATGACTGGTGGACAGCAAATGGAACAACCGATTAATGT-3'<br>Reverse1: 5'-CACTCGAGTTATACCATTGACATACCATGAGATAC-3'
Primers for SNAP-Q163A-gp17	Forward2: 5'-TATAATCTATCGCGCGCGCTCGGTAA-3'<br>Reverse2: 5'-TGGTTTTACCGAGTGCGCGCGATAGATTACAAA-3'
