# Supplementary material for: A viral genome packaging ring-ATPase is a flexibly coordinated pentamer
Source: Nat Commun. 2021 Nov 12;12:6548. doi: 10.1038/s41467-021-26800-z (PMC8589836; doi:10.1038/s41467-021-26800-z)
Supplement: Supplementary file 3 — Description of Additional Supplementary Files [file 41467_2021_26800_MOESM3_ESM.pdf]

Description of Additional Supplementary Files

File Name: Supplementary Movie 1
Description: A movie showing the proposed model of the T4 motor in complex with the DNA during packaging.
