# Supplementary material for: A viral genome packaging ring-ATPase is a flexibly coordinated pentamer
Source: Nat Commun. 2021 Nov 12;12:6548. doi: 10.1038/s41467-021-26800-z (PMC8589836; doi:10.1038/s41467-021-26800-z)
Supplement: Supplementary file 5 — Reporting Summary [file 41467_2021_26800_MOESM5_ESM.pdf]

nature research

Corresponding author(s): Prof. Venigalla Rao, Prof. Taekjip Ha, Prof. Yann Chemla

Last updated by author(s): September 26, 2021

Reporting Summary

Nature Research wishes to improve the reproducibility of the work that we publish. This form provides structure for consistency and transparency in reporting. For further information on Nature Research policies, see our Editorial Policies and the Editorial Policy Checklist.

Statistics

For all statistical analyses, confirm that the following items are present in the figure legend, table legend, main text, or Methods section.

n/a	Confirmed	
☐	☒	The exact sample size (n) for each experimental group/condition, given as a discrete number and unit of measurement
☐	☒	A statement on whether measurements were taken from distinct samples or whether the same sample was measured repeatedly
☐	☒	The statistical test(s) used AND whether they are one- or two-sided<br>*Only common tests should be described solely by name; describe more complex techniques in the Methods section.*
☒	☐	A description of all covariates tested
☐	☒	A description of any assumptions or corrections, such as tests of normality and adjustment for multiple comparisons
☐	☒	A full description of the statistical parameters including central tendency (e.g. means) or other basic estimates (e.g. regression coefficient) AND variation (e.g. standard deviation) or associated estimates of uncertainty (e.g. confidence intervals)
☐	☒	For null hypothesis testing, the test statistic (e.g. F, t, r) with confidence intervals, effect sizes, degrees of freedom and P value noted<br>*Give P values as exact values whenever suitable.*
☒	☐	For Bayesian analysis, information on the choice of priors and Markov chain Monte Carlo settings
☒	☐	For hierarchical and complex designs, identification of the appropriate level for tests and full reporting of outcomes
☒	☐	Estimates of effect sizes (e.g. Cohen's d, Pearson's r), indicating how they were calculated

Our web collection on statistics for biologists contains articles on many of the points above.

Software and code

Policy information about availability of computer code

Data collection	A customized software for single molecule fluorescence, which can be downloaded from the Ha Group website at http://ha.med.jhmi.edu/resources/. A custom Labview program and MATLAB for optical trapping experiment. Additional software is also available at : https://github.com/Ha-SingleMoleculeLab
Data analysis	MATLAB (versions >R2016), Visual Studio (Dev10), OriginPro9.2, IDL6.1, ImageJ2.1 and custom software for single molecule fluorescence which can be downloaded from the Ha Group website at http://ha.med.jhmi.edu/resources/. Additional software is also available at : https://github.com/Ha-SingleMoleculeLab

For manuscripts utilizing custom algorithms or software that are central to the research but not yet described in published literature, software must be made available to editors and reviewers. We strongly encourage code deposition in a community repository (e.g. GitHub). See the Nature Research guidelines for submitting code & software for further information.

Data

Policy information about availability of data

All manuscripts must include a data availability statement. This statement should provide the following information, where applicable:

- Accession codes, unique identifiers, or web links for publicly available datasets
- A list of figures that have associated raw data
- A description of any restrictions on data availability

The data are available upon request to the corresponding authors. No restrictions exist on the data availabilty.The reporting summary is also available with the manuscript.

Field-specific reporting

Please select the one below that is the best fit for your research. If you are not sure, read the appropriate sections before making your selection.

- [x] Life sciences
- [ ] Behavioural & social sciences
- [ ] Ecological, evolutionary & environmental sciences

For a reference copy of the document with all sections, see nature.com/documents/nr-reporting-summary-flat.pdf

Life sciences study design

All studies must disclose on these points even when the disclosure is negative.

Sample size	For each single molecule fluorescence experiment case, 244 to 1251 individual trajectories were analyzed, which were taken from replicate experiments (described below). These number of trajectories are considered more than stastitically sound for reliable interpretation, as observed in all single-molecuule experiments, also discussed in Roy et al. Nature Methods v507–516 (2008) and Colquhoun, D. & Hawkes, A.G. The principles of the stochastic interpretation of ion-channel mechanism. in Single Channel Recording. (eds. B. Sakmann & E. Neher) 397–482 (Plenum Press, New York, 1995).<br>For optical trapping experiments, 44 and 62 individual packaging traces of WT motors and ISD motors respectively were analyzed, which were sufficient to generate reliable mean values and standard deviations.
Data exclusions	No data was systematically excluded in single molecule fluorescence experiments. Some data was excluded from analysis in optical trapping experiment if unexpected tether was observed according to the criteria described in Methods.
Replication	The single molecule fluorescence experiments were replicated n=2 to 5 times and consistent results were obtained. The optical trapping experiments were repeated with multiple (>100) complexes and all findings were reproducible.
Randomization	The samples were not allocated into separate experimental groups. Therefore, the randomization was not necessary.
Blinding	Our investigation did not include subjective investigation/interpretation and as such our investigation could not be infuenced by bias. Plus, we did not do sample allocation or randomization, so the blinding was not relevant for our experiments.

Reporting for specific materials, systems and methods

We require information from authors about some types of materials, experimental systems and methods used in many studies. Here, indicate whether each material, system or method listed is relevant to your study. If you are not sure if a list item applies to your research, read the appropriate section before selecting a response.

Materials & experimental systems

n/a	Involved in the study	
[ ]	[x]	Antibodies
[x]	[ ]	Eukaryotic cell lines
[x]	[ ]	Palaeontology and archaeology
[x]	[ ]	Animals and other organisms
[x]	[ ]	Human research participants
[x]	[ ]	Clinical data
[x]	[ ]	Dual use research of concern

Methods

n/a	Involved in the study	
[x]	[ ]	ChIP-seq
[x]	[ ]	Flow cytometry
[x]	[ ]	MRI-based neuroimaging

Antibodies

Antibodies used	Bacteriophage T4-specific polyclonal antibodies were raised either in mice or in rabbits in authors' laboratory.
Validation	Binding of T4-antibodies to protein G-coated microspheres or passivated surfaces was optimized by using different concentrations of the interacting components. Some of these data are also described in the manuscript.
